# Impaired allocentric spatial processing in posttraumatic stress disorder

**DOI:** 10.1016/j.nlm.2015.01.007

**Published:** 2015-03

**Authors:** Kirsten V. Smith, Neil Burgess, Chris R. Brewin, John A. King

**Affiliations:** a Royal Holloway, University of London, Egham, Surrey, United Kingdom; b Institute of Cognitive Neuroscience and Institute of Neurology, University College London, Gower Street, London, United Kingdom; c Research Department of Clinical, Educational and Health Psychology, University College London, Gower Street, London, United Kingdom; d Department of Experimental Psychology, University of Oxford, Oxford, United Kingdom

**Keywords:** Trauma, PTSD, Hippocampus, Allocentric memory, Spatial memory

## Abstract

A neurobiological dual representation model of PTSD proposes that reduced hippocampus-dependent contextual processing contributes to intrusive imagery due to a loss of control over hippocampus-independent sensory and affective representations. We investigated whether PTSD sufferers show impaired allocentric spatial processing indicative of reduced hippocampal functioning.

Trauma-exposed individuals with (*N* = 29) and without (*N* = 30) a diagnosis of PTSD completed two tests of spatial processing: a topographical recognition task comprising perceptual and memory components, and a test of memory for objects’ locations within a virtual environment in which the test is from either the same viewpoint as presentation (solvable with egocentric memory) or a different viewpoint (requiring allocentric memory).

Participants in the PTSD group performed significantly worse on allocentric spatial processing than trauma-exposed controls. Groups performed comparably on egocentric memory and non-spatial memory for lists of objects. Exposure to repeated incident trauma was also associated with significantly worse spatial processing in the PTSD group.

Results show a selective impairment in allocentric spatial processing, implicating weak hippocampal functioning, as predicted by a neurobiological dual representation model of PTSD. These findings have important clinical implications for cognitive therapy.

## Highlights

• PTSD sufferers/trauma controls completed tests of hippocampus-dependent processing.
• The PTSD group were selectively impaired on allocentric spatial processing.
• Other spatial and non-spatial measures were comparable across groups.
• Multiple traumas resulted in poorer performance on all spatial measures.
• Viewpoint alterations to the trauma memory may be implicated in cognitive therapy.

## 1 Introduction

Posttraumatic stress disorder (PTSD) is a debilitating condition in which sufferers experience highly distressing intrusive memories, consisting of vivid sensory recollections of the original event (American Psychiatric Association, 2000; Brewin, 2003; Ehlers & Clark, 2000; Ehlers, Hackmann, & Michael, 2004). Structural and functional neuroimaging techniques have been used to investigate the integrity of the hippocampus in PTSD and, while differences have been observed relative to controls (Bremner, 2001), any corresponding cognitive dysfunction reliant on the hippocampus remains unclear. The importance of the hippocampus in the aetiology of PTSD has been confirmed by a study in which identical twins were discordant for combat exposure. The study revealed that veterans who developed PTSD had a smaller hippocampus than those without PTSD (Gilbertson et al., 2002). Crucially, the same pattern of diminution was seen in the stay-at-home twins of the veterans who developed PTSD compared with the stay-at-home twins of the no-PTSD veterans, suggesting that reduced hippocampal volume may pre-date trauma exposure and may influence the subsequent development of PTSD afterwards. However the hippocampus is also sensitive to stress-related atrophy resulting from the stress hormone cortisol acting on glucocorticoid receptors (Watanabe, Gould, & McEwen, 1992), and it has been suggested that a kindling effect exists in which experiencing prior trauma increases the impact of each additional trauma (Seidler & Wagner, 2006), implying that impaired hippocampal function may be both a risk factor for and a consequence of the condition.

To understand the hippocampal role in PTSD, a consideration of its normal function is helpful. While essential for healthy memory encoding and retrieval (Cohen & Eichenbaum, 1993; Scoville & Milner, 1957; Squire & Zola-Morgan, 1991), within the spatial domain the hippocampus is specifically implicated in allocentric representation (i.e. representation of the locations of environmental features relative to each other) as opposed to egocentric representation (i.e. representation of locations relative to the viewer), see (Burgess, Maguire, & O’Keefe, 2002; Byrne, Becker, & Burgess, 2007; O’Keefe and Nadel, 1978). Tasks that require allocentric representations of spatial layout or object location have been found to be specifically sensitive to hippocampal damage (Abrahams et al., 1999; Hartley et al., 2007; Holdstock et al., 2000; King, Burgess, Hartley, Vargha-Khadem, & O’Keefe, 2002; Lee et al., 2005; Spiers, Burgess, Hartley, Vargha-Khadem, & O’Keefe, 2001), consistent with a more general hippocampal role in flexible-relational and contextual representation beyond the spatial domain (Cohen & Eichenbaum, 1993; Cohen & Eichenbaum, 1993; Eichenbaum & Cohen, 1988). Previous studies have looked at PTSD and memory for associations between paired stimuli, reporting inconsistent findings, with some studies demonstrating impairments (Golier et al., 2002; Guez et al., 2011; Levy-Gigi et al., 2012; Yehuda et al., 2007), and others not (Bremner et al., 2003; Geuze, Vermetten, Ruf, de Kloet, & Westenberg, 2008; Gurvits et al., 1996; Shin et al., 2004).

It has been suggested that, under conditions of extreme stress due to trauma, hippocampal processing is impaired and PTSD arises from disrupted encoding of the context of the event, relative to spared affective/sensory representations of the traumatic content, mediated by the amygdala and sensory cortices (Brewin, Gregory, Lipton, & Burgess, 2010; Nadel & Jacobs, 1998). In this view, hippocampally-mediated allocentric spatial representations form an important part of the contextual representations that are disrupted in PTSD (Brewin et al., 2010). A number of recent studies have attempted to assess allocentric spatial processing in PTSD as a way of investigating hippocampal function (Gilbertson et al., 2007; Tempesta, Mazza, Iaria, De Gennaro, & Ferrara, 2012).

Gilbertson et al. (2007) used two tasks requiring the mental manipulation of spatial configurations on a folded piece of paper that is unfolded, or on the faces of a cube that is rotated, to test the identical twins discordant for combat exposure described above. Impairments were found in the veterans with PTSD as well as their stay-at-home twins. Despite being described as “historically” allocentric, it is unclear whether participants performed this task by mentally rotating their own viewpoint requiring allocentric representations (hippocampally dependent), or mentally rotated the object in front of them using an egocentric frame of reference. The latter is unlikely to require hippocampal processing (Farah & Hammond, 1988; King, Burgess, Hartley, Vargha-Khadem, & O’Keefe, 2002), as opposed to tasks that require representation of a spatial layout within which one’s own viewpoint is varied (Burgess, 2006; King, Burgess, Hartley, Vargha-Khadem, & O’Keefe, 2002). The impairments seen in this study showed a correlation with hippocampal volume, but it was not clear whether this relationship was additional to that expected due to the observed difference in hippocampal volume between the PTSD-related and control groups (Gilbertson et al., 2007). We therefore proposed to investigate the relationship between PTSD and performance on cognitive tasks that are more clearly allocentric in nature and specifically associated with hippocampal function *a priori*.

Tempesta et al. (2012) investigated memory on a virtual reality town navigation paradigm. Participants’ starting point across trials varied, requiring them to access allocentric representations of the cognitive map to perform accurately. Results showed no significant differences in accuracy of memory between the PTSD group and controls. However, groups were allowed as long as needed to build a cognitive map of the town, defined as accurately locating landmarks within the town on a two dimensional map. The PTSD group took twice as long to do this as controls, suggestive of an impairment in the ability to learn and/or utilize allocentric representations. Another consideration is that this task is unable to isolate allocentric cognitive-map based processing from egocentric landmark based processing, both of which are likely employed simultaneously by participants during navigation. The neuropsychologically validated tasks employed in the present study have been designed to directly compare egocentric and allocentric memory and topographical allocentric processing, allowing hippocampally-dependent specificity.

A further virtual reality navigation study looked at PTSD and unexposed controls and found reduced hippocampal activation predicted increased severity of PTSD in the absence of any memory differences (Astur et al., 2006). Allocentric memory impairments would have been predicted in these two navigation studies in line with animal studies, but small sample sizes of less than 25 may have contributed to null findings (Astur et al., 2006; Tempesta et al., 2012). Neither of these studies measured general visuo-spatial ability. Gilbertson et al. (2007) finding that general visualization ability was associated with performance on a visuo-spatial working memory task indicates that it is important to carefully control visuo-spatial processes when designing tests to specifically investigate allocentric memory as an indicator of hippocampal functioning in PTSD (Gilbertson et al., 2007).

Here we tested the hypothesis that allocentric spatial processing and memory would be specifically impaired in patients with PTSD compared to a matched control group. We used two tasks previously shown to be impaired in patients with hippocampal damage: memory for object locations tested from a shifted point of view compared to encoding (King, Burgess, Hartley, Vargha-Khadem, & O’Keefe, 2002; King, Trinkler, Hartley, Vargha-Khadem, & Burgess, 2004), and processing of the topographical layout of spatial scenes (Hartley et al., 2007; Lee et al., 2005). We tested the specificity of the impairment by including control tasks of egocentric spatial memory (memory for object locations tested from the same viewpoint as encoding) and non-spatial memory for lists of objects. We tested the generality of the effect by including individuals with PTSD arising from single vs. multiple events.

## 2 Methods and materials

### 2.1 Participants

Thirty trauma-exposed volunteers without PTSD aged 18–65 years (19 men) were recruited via the University College London volunteer system. Twenty-nine individuals diagnosed with PTSD by a clinical psychologist (15 men) were recruited from three specialist treatment centres in London. Non-clinical participants did not meet criteria for PTSD as assessed by a score below 11 on the Posttraumatic Stress Diagnostic Scale (PDS, Foa, 1995). Non-clinical participants were screened for current Axis I disorders. One non-clinical participant was excluded for scoring in the moderate range for depression on the Beck Depression Inventory-Version II (BDI-II, Beck, Steer, & Brown, 1996), while another met criteria for trichotillomania, but her scores were typical and were included in analyses. No participants met criteria for current substance and alcohol dependence or suffered a head injury in the last year. Five participants were taking medications that had the potential to interfere with hippocampally dependent memory processes (GABA agonists, benzodiazepines, and opiates). However, the means and standard deviations of this group were similar to the no-medication group and were included in analyses. The study was approved by the Leeds and Humberside NHS ethics committee.

### 2.2 Procedure

Participants completed clinical measures for PTSD (PDS, Foa, 1995) and depression (BDI-II, Beck et al., 1996) followed by a brief (12-item) measure of general visuospatial ability included as a screening tool for learning disabilities (Raven’s Advanced Progressive Matrices: RAPM, Set I; Raven, 1941). The RAPM is a standardised culturally neutral neuropsychological test of non-verbal abstract reasoning and visual-spatial problem-solving abilities and consists of visual pattern matching and analogy problems, requiring the subject to conceptualize spatial, design, and numerical relationships. Information about whether participants’ index trauma was a single or a repeated event was also recorded. A single event was categorised as one occasion in which the participant had experienced actual or threatened death or serious injury to themselves or others or there was a threat to the physical integrity of themselves or others. The event also had to evoke the subjective experience of intense fear, helplessness or horror (American Psychiatric Association, 2000). Repeated event trauma was categorised if there were multiple occasions of the above criteria. An example of a single event trauma might be an assault while a repeated event trauma might be domestic violence. The first phase of the non-spatial object list-learning was then administered followed by either the Four Mountains Task or the Town Square Task. Participants then completed the second phase of the object list-learning task, the remaining allocentric processing task, and finally recall of the object list.

### 2.3 Assessments

#### 2.3.1 Non-spatial object list-learning

This task was adapted from a paradigm by Hupbach et al. (2007) in which two baskets of objects are presented sequentially to participants separated by a time period. Basket I contains 20 household items, and Basket II contains 14 new unique items and six items from the previous basket (three at the start of the list and three at the end). Items are presented one by one and participants instructed to name the item and place it behind them out of sight. Following this, participants are asked to order the items by length on a table and are timed. Naming and sorting was employed to encourage participants to notice both the conceptual (name) and perceptual (length) features of the items. Prior to the presentation of Basket II participants are reminded about the first basket and told that the same procedure is to be adopted. The spatial processing tasks were administered between basket presentation and item recall. At the end of the session participants were asked to recall as many items as possible. Total number of items recalled and the basket of origin (I or II) was recorded. This task was included to investigate hypotheses relevant in another line of research. Its inclusion in this study acted as a non-spatial memory control to determine whether traumatised individuals with and without PTSD differed on another form of memory such as object list-learning.

#### 2.3.2 The Four Mountains Task (Hartley et al., 2007) – allocentric processing

##### 2.3.2.1 Topographical perception

**Fig. 1:**
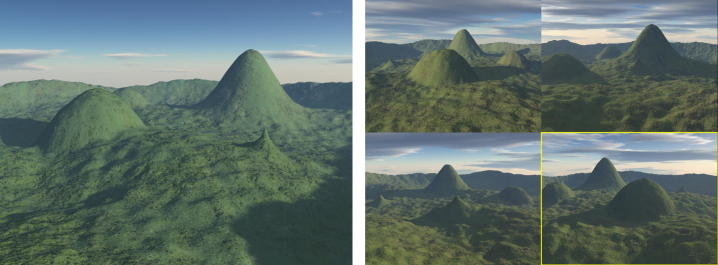
The Four Mountains Task. Left panel: a typical example of a stimulus image. Right panel: four forced-choice response items. Each shows a different arrangement of topography, one of which is the stimulus shown from a different position. The correct response is highlighted for clarity in this figure only.

Participants are presented with a “sample” landscape image, and simultaneously with a four-alternative choice of landscape scenes arranged in a 2 by 2 grid on the facing page of the A4 test booklet (Fig. 1). All four alternative responses are rendered under the same weather and lighting conditions as each other, but with different conditions and viewpoint from the sample image. The task is to identify the target image that matches the topography of the sample image. Items were presented for a maximum of 60 s and participants were prompted for a response after 30 s. Responses correct are scored out of 15 items.

##### 2.3.2.2 Topographical memory

The topographical memory task is the same as the topographical perception task, except the sample image is presented in isolation for approximately 8 s. The page of the booklet is then turned to a blank page for approximately 2 s followed by a page with a four-way choice in which they had 30 s to provide an answer.

The four alternative responses are generated for each sample landscape in the same way as the perceptual task, but in order to match difficulty with the perceptual version of the task the foils are made slightly more distinctive from the target. Responses correct are scored out of 15 items.

#### 2.3.3 The Town Square Task (King et al., 2002, 2004) – allocentric spatial memory

**Fig. 2:**
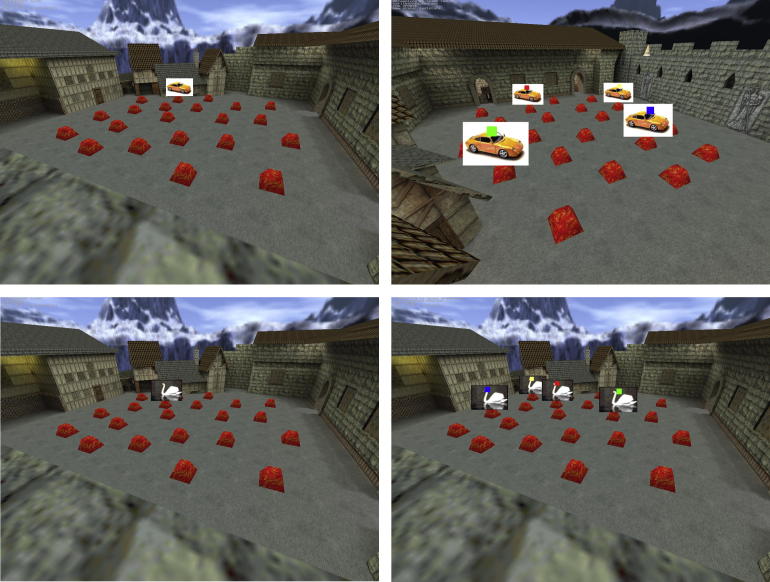
The Town Square Task. Left panels show learning items, right panels show the memory test, with one item in the original location and three foil copies in random locations. Upper panels show a shifted-view item at learning and test, lower panels show a same-view item. Colors are used for participants to indicate their chosen response.

Viewpoint-dependent memory was assessed through the use of a virtual environment (VE) observed on a laptop, consisting of a courtyard surrounded by visually distinct buildings (Bisby, King, Brewin, Burgess, & Curran, 2010). Participants could navigate along two of the VE perimeter walls at rooftop level. Within the courtyard, 21 placeholders were randomly distributed and used for the presentation of test stimuli. Presentation and test used two viewpoints located in opposite corners of the courtyard, involving a rotation of 140° in viewing orientation when moving from one view to the other. Participants were required to navigate toward one of the marked presentation locations and on contact their view was automatically adjusted to a standard view of the courtyard with all placeholders visible (Fig. 2).

At presentation, images of everyday objects appeared one at a time on placeholders within the VE for 3 s each, with a 1-s inter-stimulus interval. The number of objects presented in each trial was counterbalanced between two list lengths (*n* = 3, *n* = 6) to reduce predictability and development of potential mnemonic strategies. Participants were instructed to remember the location of each object. After each trial, memory was tested either from the same viewpoint as presentation or from the other viewpoint. Viewpoint at test was counterbalanced, and the presentation order of viewpoint and list length were randomized. Memory for object locations was tested in a random order with each object presented on the original placeholder and three foils (copies of the object) on other placeholders. Each object image included a colored square superimposed on it and participants were required to press the corresponding colored key on the keyboard to identify their chosen location, participants were given as long as needed to answer. Importantly performance between same-view and shifted-view conditions are approximately difficulty matched by restricting the foils in the same-view condition to the nearest five locations to the target, while spreading them evenly over all other locations in the shifted-view condition, resulting in comparable performance across conditions in control participants (King et al., 2004). Participants received an overall percentage correct score for same and shifted-view memory conditions collapsed across set size. We assume that the same-view task can be solved by egocentric processes acting on the scenes at encoding and test, whereas the shifted-view task requires allocentric processing (i.e. viewpoint-independent knowledge of locations) in addition to these egocentric processes. Thus the same-view task can be used to control for differences in egocentric processing when analysing the allocentric processing required by the shifted-view task (Bisby et al., 2010).

### 2.4 Statistical analysis

All statistical analyses were performed with SPSS version 20 (SPSS, IBM Corp. in Armonk, NY). Allocentric spatial processing on the Four Mountains Task was analysed using a mixed factorial analysis of variance (ANOVA), with group as a between-participant factor (no-PTSD vs. PTSD) and condition (perception vs. memory) as the within-participant factor. The potential influence of demographic and cognitive group differences on overall allocentric processing (perception + memory) were investigated with multiple regression. The same ANOVA procedure was adopted for the Town Square Task [group (no-PTSD vs. PTSD) × viewpoint (same-view vs. shifted-view)]. The same-view scores were subtracted from the shifted-view scores to isolate allocentric memory performance controlling for (egocentric) spatial memory performance, and this variable was employed as a dependent variable within similar multiple regressions. The non-spatial object learning task was also investigated with ANOVA [group (no-PTSD vs. PTSD) × basket (I vs. II)]. To assess any contribution of trauma type on spatial memory in the PTSD group, a repeated measures ANCOVA (Four Mountains – Memory total, Perception total) was conducted with trauma type (single vs. repeated event) as the between-subjects factor and the PDS total score as the covariate to control for differences in PTSD severity. The same procedure was adopted for the Town Square Task (same-view total, shifted-view total). As above multiple regression was used to assess the influence of confounding factors, however, in order to get a fuller picture of the effects of repeated traumatisation on overall cognitive functioning both hippocampally dependent and independent, an overall spatial processing score was calculated for the Four Mountains (Perception + Memory) and the Town Square Task (same-view total + shifted-view total).

## 3 Results

### 3.1 Demographic data

As shown in Table 1, groups did not differ significantly on demographic variables although there was a trend for the non-PTSD group to have had more years of education. The PTSD group also had significantly more exposure to repeated event trauma. Groups showed the expected differences on all clinical measures. In addition the PTSD group scored significantly lower on the Progressive Matrices test of non-verbal intellectual functioning.

### 3.2 Allocentric spatial processing (Four Mountains Task)

**Fig. 3:**
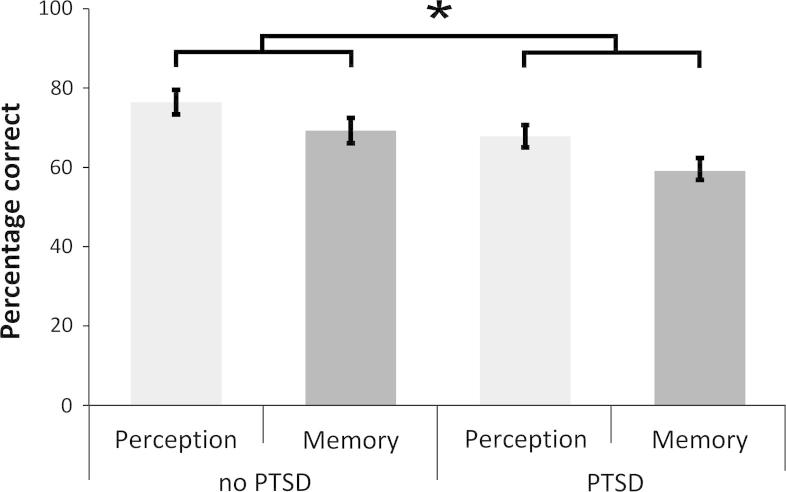
Performance on the Four Mountains Task. Asterix indicates a significant (*P* < 0.05) main effect of group (PTSD vs. control), no other comparisons were significant. Error bars represent two standard errors.

A 2 × 2 mixed model ANOVA did not show an interaction of condition and group [*F*(1, 55) = .13, *p* = .72], reflecting a similar pattern of performance across perception and memory by both groups (Fig. 3). There was a main effect of condition [*F*(1, 55) = 12.77, *p* = .001, *η_p_*^2^ = .19], reflecting the overall higher performance on allocentric perception than memory by both groups. There was also a significant main effect of group, [*F*(1, 55) = 6.18, *p* = .02, *η_p_*^2^ = .10], reflecting a poorer performance on overall allocentric spatial processing of the PTSD group compared with the no-PTSD group. See Fig. 3.

Potentially confounding variables age, education and Raven’s Advanced Progressive Matrices were entered into a hierarchical regression at step 1, a single overall allocentric processing score was calculated (Perception + Memory) and entered as the dependent variable and Group at step two. Confounding factors explained a significant amount of variance in allocentric processing (*F*(3, 53) = 5.21, *p* = .003; *R*^2^ = .23, adjusted *R*^2^ = .18). At the second step Group did not account for a significant additional proportion of the variance (*F*(1, 52) = 1.34, *p* = .253; *R*^2^ = .25, adjusted *R*^2^ = .19), and in the final equation only the RAPM provided a unique contribution to allocentric processing (*b* = .87, *t*(52) = 3.00, *p* = .004). Therefore it is possible that variation in the more general intellectual and visuospatial processing tapped by RAPM contributed to the group differences in performance on the allocentric spatial task.

### 3.3 Allocentric spatial memory (Town Square Task)

**Fig. 4:**
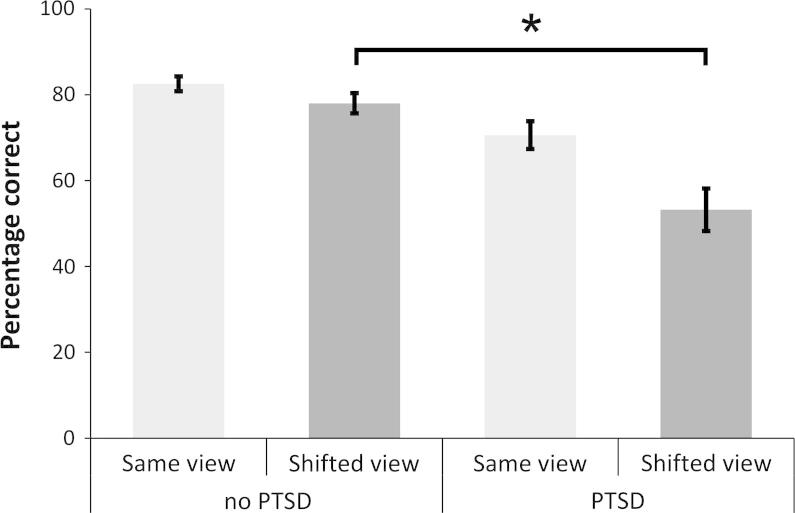
Performance on egocentric and allocentric spatial memory in the Town Square task, by group (∗*P* = .01).

A 2 × 2 mixed factorial analysis showed a significant interaction of group and viewpoint [*F*(1, 55) = 6.68, *p* = .01, *η_p_*^2^ = .11], reflecting the poorer performance by the PTSD group on the shifted-view compared to same-view spatial memory task (see Fig. 4). There was a main effect of viewpoint [*F*(1, 55) = 105.69, *p* < .001, *η_p_*^2^ = .66], reflecting the poorer performance of both groups in the allocentric (i.e. shifted-view) condition. The main effect of group was also significant [*F*(1, 55) = 5.10, *p* = .03, *η_p_*^2^ = .09] indicating that the PTSD group performed significantly worse than the no-PTSD group on overall spatial memory. Post hoc t-tests confirmed that groups did not differ on same-view spatial memory [*t*(55) = 1.48, *p* = .14] but the PTSD group displayed a selective impairment on shifted-view spatial memory [*t*(55) = 2.58, *p* = .01].

A shifted-view minus same-view memory score was used as the dependent variable in the same hierarchical regression described previously to investigate predictors of allocentric memory when egocentric memory was controlled. The Town Square Task was designed with this approach in mind; all aspects of the trials are identical across conditions other than the viewpoint change, hence egocentric performance represents baseline ability. At step 1 age, education and RAPM did significantly influence allocentric processing (*F*(3, 53) = 3.40, *p* = .02; *R*^2^ = .16, adjusted *R*^2^ = .11). Results revealed a significant effect of Group (PTSD performing worse) at step two (*F*(1, 52) = 4.38, *p* = .04; *R*^2^ = .23, adjusted *R*^2^ = .17), and in the final equation only Group (*b* = *−*492.15, *t*(52) = −2.09, *p* = .04) and age (*b* = *−*22.33, *t*(52) = −2.54, *p* = .01) provided a unique contribution to allocentric processing. Therefore while the differential performance of the two groups that is specific to allocentric memory, controlling for egocentric memory, is in part related to age, PTSD remains a significant predictor.

### 3.4 Non-spatial object list-learning

A 2 × 2 [group (no-PTSD vs. PTSD) × basket (I vs. II)] mixed factorial analysis did not reveal a significant interaction of group and basket [*F*(1, 55) = .55, *p* = .46, *η_p_*^2^ = .01], reflecting a similar pattern of performing across baskets between groups. There was a main effect of basket [*F*(1, 55) = 28.34, *p* < .001, *η_p_*^2^ = .34] – both groups recalled more items from basket II – but no main effect of group [*F*(1, 555) = .03, *p* = .87, *η_p_*^2^ = .00].

### 3.5 Trauma type and allocentric processing

#### 3.5.1 Demographic data

As shown in Table 2, the single event trauma group were significantly younger with fewer months since their traumatic event, and there was a trend for them to score higher on the RAPM. Groups showed no differences on any clinical measure. There was no effect of trauma type on non-spatial object list-learning and as such only allocentric spatial processing and memory were included in these analyses.

#### 3.5.2 Allocentric spatial processing (Four Mountains Task)

We investigated the relationship between trauma type and spatial processing in the PTSD group. The PDS (Posttraumatic Stress Diagnostic Scale, Foa, 1995) total was entered as a covariate into a repeated measures ANCOVA with trauma type as the between subject factor and the two subtests of the Four Mountains as the dependent variables. The same procedure was adopted for the subtests of the Town Square Task. Following this, in order to assess the contribution of the potentially confounding factors of age, education, and RAPM, trauma type replaced group in the hierarchical regression already described.

The repeated trauma group performed significantly worse on both allocentric perception and memory measures of the Four Mountains (*F*(1, 26) = 10.77, *p* = .003, *η_p_*^2^ = .29). There were no significant main effects of the PDS subscales on allocentric processing, nor were there any significant interactions with trauma type.

Regression analyses revealed that age, education and RAPM did significantly influence allocentric processing (*F*(3, 25) = 3.72, *p* = .02; *R*^2^ = .31, adjusted *R*^2^ = .22) at step 1. Results revealed a significant effect of Trauma Type (Repeated trauma performing worse) at step two (*F*(1, 24) = 5.83, *p* = .02; *R*^2^ = .44, adjusted *R*^2^ = .35), and in the final equation only Trauma Type (*b* = *−*3.45, *t*(24) = −2.41, *p* = .02) and RAPM (*b* = .81, *t*(24) = 2.78, *p* = .03) provided a unique contribution to allocentric processing on the Four Mountains.

#### 3.5.3 Spatial memory (Town Square Task)

Similarly, the repeated trauma group performed significantly worse on both same-view and shifted-view memory measures in the Town Square Task (*F*(1, 26) = 8.01, *p* = .009, *η_p_*^2^ = .23) after controlling for PDS total, which did not significantly affect scores. There was no interaction of trauma type with same vs. shifted-view processing.

Given the overall drop in performance on the Town Square Task, an overall spatial processing score (same-view + shifted-view) was computed and entered as the dependent variable into the multiple regression.

At step 1 age, education and RAPM significantly influenced allocentric processing (*F*(3, 25) = 5.23, *p* = −.01; *R*^2^ = .39, adjusted *R*^2^ = .31). Results of Trauma Type at step two approached statistical significance (*F*(1, 24) = 4.10, *p* = .05; *R*^2^ = .48, adjusted *R*^2^ = .39) and in the final equation only RAPM (*b* = 5.73, *t*(24) = 2.52, *p* = .02) provided a unique contribution to overall spatial processing, while the contribution of Trauma Type (*b* = *−*18.48, *t*(24) = −2.03, *p* = .05) approached significance.

## 4 Discussion

### 4.1 Allocentric processing

Previous studies investigating how aspects of memory implicating the hippocampus are affected in PTSD have revealed inconsistent results with some finding impairment (Golier et al., 2002; Guez et al., 2011; Yehuda et al., 2007) and some not (Bremner et al., 2003; Geuze et al., 2008; Gurvits et al., 1996; Shin et al., 2004). We tested a more specific hypothesis derived from the revised dual representation theory of PTSD (Brewin et al., 2010) and consistent with earlier research (Gilbertson et al., 2007; Nadel & Jacobs, 1998), namely that PTSD sufferers would have a selective impairment in allocentric spatial processing. The tasks employed assessed allocentric spatial processing of the topographical layout of environmental scenes in both perception and memory, as well as memory for locations of individual objects. Importantly, these different tasks are proposed to be underpinned by the same cognitive operations reliant on the hippocampus (Burgess, Maguire, & O’Keefe, 2002). The results revealed a selective deficit in allocentric spatial processing in PTSD sufferers compared with matched traumatized controls, consistent with and extending previous findings (Gilbertson et al., 2007; Hartley et al., 2007; King et al., 2002; Tempesta et al., 2012). Further analysis on trauma type in the PTSD group revealed that those with repeated event trauma performed significantly worse on all spatial memory measures, rather than showing a specific allocentric deficit.

### 4.2 Allocentric spatial memory

The memory impairments reported in the PTSD group were specific to the types of allocentric spatial processing which have been shown to be hippocampally-dependent (Hartley et al., 2007; King et al., 2002). Importantly these individuals did not show a general cognitive impairment, performing below ceiling and similarly to controls on remembering lists of objects and egocentric spatial memory. However, in line with some previous research (Gilbertson et al., 2007), they scored lower than the no-PTSD group on a brief measure of general visuo-spatial ability. Thus, although the Four Mountains task is well-established as a test of allocentric spatial processing (Hartley et al., 2007), in this particular dataset we cannot rule out the possibility that general visuo-spatial ability contributed to the group difference we found.

In contrast, the Town Square task group difference remained statistically significant after controlling for differences in general visuospatial ability. This task is uniquely useful as its design enables it to better distinguish the selective contribution of allocentric spatial processing after controlling for egocentric spatial processing. Group differences on this task could not be accounted for by general visuo-spatial ability, presumably because they load equally on both allocentric and egocentric components of the task. Future research could profitably investigate other measures of intellectual functioning that load minimally on general verbal and non-verbal IQ as a way of better isolating the relationship between PTSD and hippocampally-dependent cognitive processes. The results can be tentatively explained by the functional and anatomical abnormalities in the hippocampal region that are typically associated within PTSD (Bremner et al., 2003; Karl et al., 2006; Shin et al., 2004). However, further imaging research is needed to assess the potential contribution of other structures associated with allocentric processing and PTSD (Gomez, Cerles, Rousset, Rémy, & Baciu, 2014; Pitman et al., 2012).

### 4.3 Trauma exposure

There is a debate within the literature about whether a reduction in hippocampal size and function results from exposure to PTSD-related stress or is a risk factor for PTSD development (Bonne et al., 2001; Carrion et al., 2001; De Bellis et al., 2002; Fennema-Notestine, Stein, Kennedy, Archibald, & Jernigan, 2002; Gilbertson et al., 2002). For example, animal studies have shown degradation of the CA3 neurons in the hippocampus as a result of repeated exposure to stress (Watanabe et al., 1992). The present study showed that in the PTSD group, repeated exposure to trauma was associated with poorer performance on overall spatial memory function, irrespective of whether from same or shifted view. After controlling for confounding factors age, education and visuospatial processing repeated traumatisation remained a significant predictor of reduced allocentric processing on the Four Mountains Task. These results are in line with a previous longitudinal study (Perez & Widom, 1994) that found that that child abuse and neglect represented significant risk factors for poor long-term intellectual and academic outcomes in young adulthood compared to matched controls. We did not however find any association between trauma type and the specific deficit hypothesized to underlie vulnerability to PTSD, suggesting that this risk factor is independent of environmental exposure to trauma.

### 4.4 Clinical implications

These data have important implications for trauma-focused psychological therapy for PTSD (Ehlers & Clark, 2000; Grey, Holmes, & Brewin, 2001), a process in which sufferers are asked to relive their traumatic event in imagery and update inappropriately negative appraisals. The standard approach to reliving involves imagining the traumatic scene happening in front of their eyes, i.e. retrieving an egocentric representation, whereas the revised dual representation theory proposes that imagining it from a different perspective, requiring retrieval or construction of an allocentric representation, will facilitate the recovery of contextual details and help to minimize unwanted re-experiencing. Our data suggest that individuals with PTSD may be unlikely to do this spontaneously and may find this more difficult than those without PTSD. To this extent it is likely that they would be helped by specific therapist instructions to manipulate their imagined perspective on the trauma scene. Future research should investigate the extent to which facilitation of allocentric representations within trauma memories increases contextualization and reduces intrusions.

## 5 Conclusions

In conclusion this study contributes to the literature on cognitive deficits in PTSD and is the first to indicate a selective deficit in hippocampal-dependent allocentric spatial memory. These findings offer support for a theoretical perspective that suggests new therapeutic possibilities from employing allocentric representations within psychological therapy for PTSD.

## Figures and Tables

**Table 1:** Means (SDs) for demographic and clinical data.

	PTSD (*n* = 29)	No-PTSD (*n* = 29)	*t*, *χ*^2^	*p*
Age in years *M* (SD)	40.76 (11.10)	36.75 (13.98)	*t* = −1.20	.23
Years of full time education	15.86 (3.86)	17.64 (3.00)	*t* = 1.93	.06
Months since index trauma	176 (196.75)	168 (153.51)	*t* = −.18	.86
Units of alcohol (per week)	7.96 (17.54)	8.37 (6.76)	*t* = .93	.36
Hours of computer games played per week	6.15 (4.45)	3.46 (7.12)	*t* = −.37	.71
Beck Depression Inventory Version-II	32.21 (15.23)	5.11 (4.82)	*t* = −9.12	<.001
Raven’s Advanced Progressive Matrices	7.44 (2.10)	9.39 (1.93)	*t* = 3.61	.001
Posttraumatic Diagnostic Scale	33.96 (11.22)	3.00 (3.55)	*t* = −14.31	<.001
Repeated trauma *N* (%)	18 (62.01)	8 (27.59)	*χ*^2^ (3, *N* = 57) = 6.61	.011

**Table 2:** Means (SDs) for demographic and clinical data by trauma type.

	Single event (*n* = 11)	Repeated event (*n* = 18)	*t*	*p*
Age in years *M* (SD)	35.45 (9.77)	44.00 (10.84)	*t* = −2.14	.04
Years of full time education	17.27 (3.61)	15.00 (3.85)	*t* = 1.58	.13
Months since index trauma	44 (38.90)	256.28 (211.88)	*t* = −4.12	.001
Units of alcohol (per week)	7.40 (4.88)	5.75 (9.42)	*t* = .37	.71
Hours of computer games played per week	4.62 (12.28)	4.89 (19.75)	*t* = −.04	.97
Beck Depression Inventory Version-II	27.36 (14.39)	35.17 (15.36)	*t* = −1.36	.19
Raven’s Advanced Progressive Matrices	8.36 (1.96)	6.89 (2.03)	*t* = 1.92	.07
Posttraumatic Diagnostic Scale	29.91 (10.68)	36.44 (11.11)	*t* = −1.56	.13
